# Inferred Ancestral Origin of Cancer Cell Lines Associates with Differential Drug Response

**DOI:** 10.3390/ijms221810135

**Published:** 2021-09-20

**Authors:** Phong B. H. Nguyen, Alexander J. Ohnmacht, Samir Sharifli, Mathew J. Garnett, Michael P. Menden

**Affiliations:** 1 Helmholtz Center Munich, Institute of Computational Biology, 85764 Neuherberg, Germany; phong.nguyen@helmholtz-muenchen.de (P.B.H.N.); alexander.ohnmacht@helmholtz-muenchen.de (A.J.O.); samir.sharifli@tum.de (S.S.); 2 Department of Biology, Ludwig-Maximilians University Munich, 82152 Martinsried, Germany; 3 Department of Mathematics, Technical University Munich, 85748 Garching, Germany; 4 Wellcome Trust Sanger Institute, Wellcome Genome Campus, Hinxton CB10 1SA, UK; mathew.garnett@sanger.ac.uk; 5 German Center for Diabetes Research (DZD e.V.), 85764 Neuherberg, Germany

**Keywords:** cancer, ancestry, high-throughput drug screen, biomarkers

## Abstract

Disparities between risk, treatment outcomes and survival rates in cancer patients across the world may be attributed to socioeconomic factors. In addition, the role of ancestry is frequently discussed. In preclinical studies, high-throughput drug screens in cancer cell lines have empowered the identification of clinically relevant molecular biomarkers of drug sensitivity; however, the genetic ancestry from tissue donors has been largely neglected in this setting. In order to address this, here, we show that the inferred ancestry of cancer cell lines is conserved and may impact drug response in patients as a predictive covariate in high-throughput drug screens. We found that there are differential drug responses between European and East Asian ancestries, especially when treated with PI3K/mTOR inhibitors. Our finding emphasizes a new angle in precision medicine, as cancer intervention strategies should consider the germline landscape, thereby reducing the failure rate of clinical trials.

## 1. Introduction

Pre-clinical studies in drug development can help to refine the target population and thus increase the success of clinical trials [1]. To this end, cancer cell lines are simplified and scalable models of human tumours, and enable the high-throughput exploration of pharmacogenetic interactions [2,3,4]. Among the largest efforts are the Genomics of Drug Sensitivity in Cancer (GDSC) project [2], Cancer Cell Line Encyclopedia (CCLE) [3] and Cancer Therapeutics Response Portal (CTRP) [4]. These efforts have screened hundreds of compounds across >1000 cancer cell lines in order to identify molecular biomarkers of drug response, thereby paving the way for precision oncology.

In the last two decades, the Cancer Genome Atlas (TCGA) [5] and International Cancer Gene Consortium (ICGC) [6] have pioneered the molecular characterisation of cancer patients. These efforts have revealed core cancer genes and their driver mutations, which are conserved in cancer cell lines (CCL) [7], and focusing on these somatic mutations assisted the identification of potential biomarkers [2]. On the other hand, germline variants have been known to influence the somatic mutational landscape of cancer tumours by changing the structures of genes and amino acid sequences, affecting the distribution of somatic mutations and causing global enrichment of mutations [8]. So far, many studies have revealed the direct effect of germline variants or their interaction with somatic mutations in predicting the outcome of patient treatments or sensitivity of cancer cell lines in high-throughput drug screens (HTSs) [9,10,11].

In contrast, genetic ancestry is mostly neglected in HTSs, although it is an established factor of risk, progression and response to treatment in several cancer types in the clinic [12,13]. Incorporating ancestry as an independent factor or covariate in drug response modelling may result in a more refined discovery of novel biomarkers, and enable us to model interactions with patient demographics.

**Figure 1:**
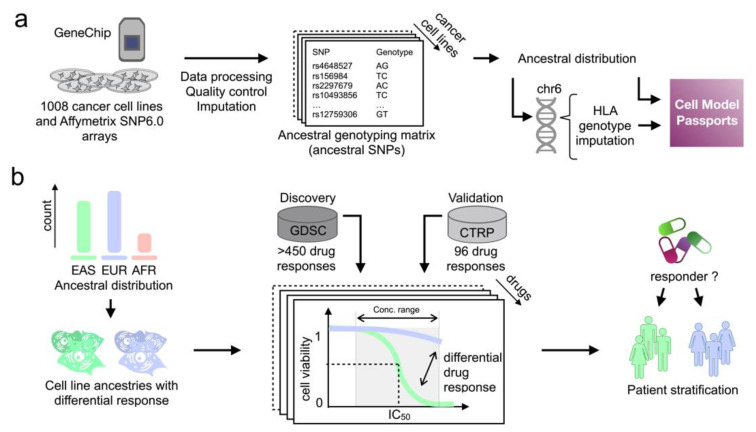
Ancestry inference pipeline and downstream analysis for differential drug response. (**a**) The analysis pipeline for retrieving an ancestral genotyping matrix by using Affymetrix SNP6.0 arrays from the GDSC CCLs, including data processing, quality control and imputation. The inferred ancestry and HLA genotypes are deployed on Cell Model Passports. (**b**) Workflow of pairwise ancestral comparisons of drug response in GDSC, validated with CTRP, generating hypotheses for patient stratifications in clinical trials based on demographics.

In this study, we leveraged the drug sensitivity profiles of >1000 molecularly characterised CCLs across >400 drugs obtained from the GDSC project for revealing ancestry-dependent pharmacogenetic interactions. First, we inferred the ancestry of the cell line panel using a Bayesian method, using a list of 100 predictive single-nucleotide polymorphisms (referred to as ancestral SNPs) [14] for inference (Figure 1a), validated in an independent HTS (CTRP). In addition, by using the cell line ancestry, we subsequently inferred the HLA genotypes of the CCLs (Figure 1a). Lastly, we identified cell line ancestries that confer drug sensitivity, ultimately revealing patient subgroups stratified by their ancestry which may show differential responses to treatments in clinical trials (Figure 1b).

## 2. Results

### 2.1. Inferred Ancestry of Cancer Cell Lines Is Conserved

Using our processing pipeline (Figure 1a), the ancestral SNPs from the GDSC genotyping data were retrieved and imputed, which was then used to calculate the ancestral probabilities of these CCLs and reveal their ancestry origin (details in Methods). Our ancestry inference pipeline is publicly available and applicable for genetically characterised human cancer models of unknown origin.

**Figure 2:**
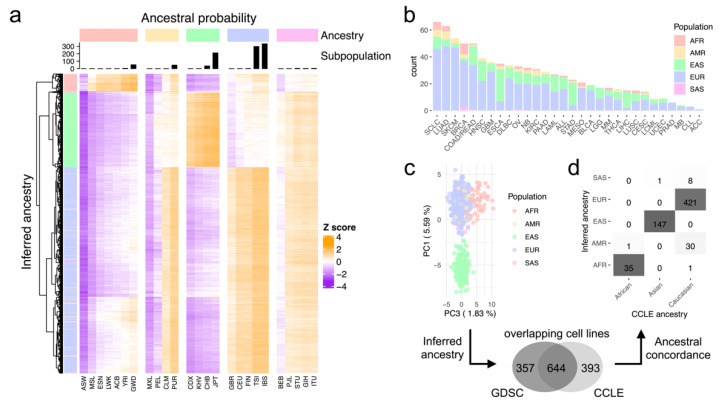
Inference of the ancestral distribution in cancer cell lines. (**a**) Heatmap of the *z*-scores for the inference of ancestry by estimating ancestral probabilities using a Bayesian method. (**b**) Distribution of inferred ancestries across cancer types. (**c**) PCA on the ancestral genotyping matrix. (**d**) Ancestral concordance between inferred ancestries of the GDSC and annotated CCLE CCLs.

The CCLs were classified in 25 subpopulations (Figure 2a), which stemmed from the ancestral origins defined by the 1000 Genomes Project (1000G) [15]. Since this level of stratification resulted in relatively small sample sizes, we summarised the subpopulations into five ancestries, namely European (EUR), East Asian (EAS), African (AFR), American (AMR) and South Asian (SAS) ancestries (Figure 2a). In this context, most cell lines were classified as EUR, followed by EAS ancestry. Particularly, EUR and EAS were assigned to 633 cells (63.1%) and 248 cells (25.0%), respectively, which together constituted the vast majority of the dataset (88.1%). The other ancestries, AFR, AMR and SAS, only accounted for 56 cells (5.7%), 47 cells (5.4%) and 10 cells (0.8%), respectively (Figure 2a). The distribution of ancestries across cancer types was conserved in many cancer types (Figure 2b). For example, 69.69% of small cell lung cancer (SCLC) were EUR (46/66 cells), in contrast with only 7.58% AMR (5/66 cells). There were a few exceptions, however, in which the vast majority of cells in a cancer type were EAS. For example, 35 CCLs were derived from oesophageal carcinoma (ESCA) samples, of which 24 CCLs were classified into EAS (68.57%). CCLs were labelled according to TCGA classification, in which a significant number of cells could not be classified into any cancer type (183/994 cells, 18.41%) (Figure 2b).

A principal component analysis using the genotype matrix showed that the cells were clustered into three distinct groups, which correspond to AFR, EAS and EUR/AMR ancestries (Figure 2c), thus highlighting a stable prediction of ancestry in CCLs.

In order to examine the validity of our method, we compared the results with a similar dataset from the CCLE project. We found 644 cell lines overlapping between the two datasets, using the shared COSMIC and DepMap cell IDs. To make our data compatible with the CCLE data, we summarised both AMR and EUR cell lines as Caucasian and EAS and SAS cell lines as Asian in the annotated GDSC set. As expected, the inferred ancestry showed very high concordance with the referenced ancestry by the CCLE. Particularly, 634 out of 644 cell lines were consistently annotated (98.4%). From the remaining ten cells, nine were CCLE Caucasian that were classified as SAS (eight cells) or AFR (1 cell) and one was an African cell line that was classified as AMR in GDSC, set by our pipeline (Figure 2d). Although based only on a subset of the data, the extremely high concordance of our results with CCLE data proved the accuracy of our analysis pipeline.

Furthermore, using the inferred ancestry information, we managed to predict the HLA genotypes of the CCLs using the HiBAG method [16] with high accuracy (Supplementary Table S1). We validated the result with 56 NCI60 cell lines; the accuracy was up to 85.7% (allowing one mismatched allele for the haplotype consisting of six loci in MHC class I and II: A, B, C, DRB1, DQB1 and DPB1). Together, ancestries and HLA genotypes of CCLs will be imported to Cell Model Passports, a catalogue of CCL annotations that serve as potential features in pharmacogenomic studies (Figure 1a).

### 2.2. Differential Drug Responses between Asian and Caucasian Cancer Cell Lines

**Figure 3:**
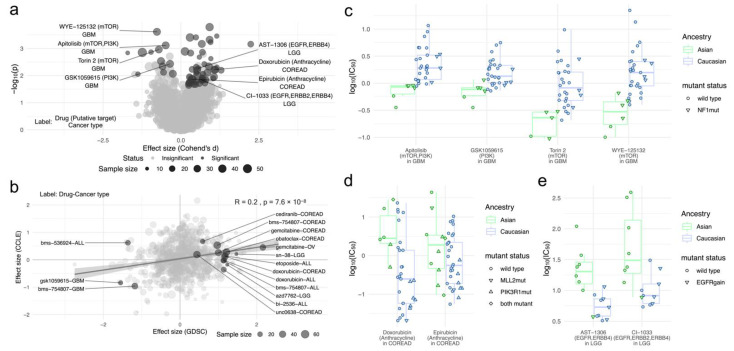
Differential drug responses between ancestral origins. (**a**) Associations between Caucasian and Asian ancestry and drug response across cancer types (<20% FDR). (**b**) Comparison of effect sizes between GDSC and CRTP. Highlighted are significant associations in GDSC, which were overlapping with CTRP. Here, the exemplified associations are higher drug sensitivity of (**c**) Asian CCLs to PI3K/mTOR inhibitors in GBM. (**d**) Caucasian CCLs to anthracyclines in COREAD and (**e**) Caucasian CCLs to TKIs in LGG.

Next, we investigated CCL ancestries which confer drug sensitivity, which may be leveraged for selecting target cohorts based on demographics. The drug response data from the GDSC project served as our discovery cohort, and CTRP was used for validation. We performed one-way ANOVA tests for investigating imbalances in drug responses across two pairwise ancestries. Comparing the largest two populations, i.e., Asian and Caucasian (Figure 1b; Methods), revealed 59 significant associations between ancestry and drug response in total across nine cancer types (Figure 3a & Supplementary Table S2, <20% FDR). Comparisons of Asian and African, as well as African and Caucasian ancestries are included in the Supplementary Materials (Supplementary Tables S3 and S4).

For validation, we investigated the overlapping screens between GDSC and CTRP (Figure 1b; Methods). Focusing on the fraction of associations between ancestry and drug response that were significant in GDSC (<20% FDR), we observed that 11 out of 16 associations from our discovery cohort displayed a consistent sign of effect size (Figure 3b). Four out of the five inconsistent validation experiments were found in acute lymphoblastic leukaemias (ALL). Across all performed tests, the Pearson correlation for effect sizes of the overlapping compounds between GDSC and CCLE was R = 0.2 (Figure 3b), suggesting reproducible associations in independent experiments.

Among the top significant associations, Asian cell lines showed higher sensitivity to PI3K/mTOR inhibitors, especially in glioblastoma (GBM). Specifically, out of all 12 associations in GBM, the cell lines were significantly sensitive to apitolisib (Cohen’s *d* = −1.73, adj. *p* = 0.06), GSK1059615 (Cohen’s *d* = −1.55, adj. *p* = 0.11), torin 2 (Cohen’s *d* = −1.87, adj. *p* = 0.06) and WYE-125132 (Cohen’s *d* = −1.91, adj. *p* = 0.04) (Figure 3c). Furthermore, the targets of those drugs (PI3K/mTOR) were enriched among Asian-sensitive associations in GBM (mTOR: adj. *p* = 0.0004, PI3K: adj. *p* = 0.01; Methods). Noticeably, the majority of these inhibitors (three out of four) target only mTOR or a combination of mTOR and PI3K, whereas GSK1059615 only targets PI3K.

Associations in which Caucasian CCLs were found to be more sensitive accounted for 47 out of 59 total significant associations (Supplementary Table S2). In fact, 16 out of 59 significant associations were found in colorectal adenocarcinoma (COREAD), all of which were found to be sensitive in Caucasian CCLs. We found that Caucasian CCLs are more sensitive to the two anthracyclines doxorubicin and epirubicin (Figure 3d). This type of drug is enriched among all significant associations in COREAD (adj. *p* = 0.09).

Furthermore, we identified that Caucasian CCLs in low-grade glioma (LGG) were more sensitive to irreversible tyrosine kinase inhibitors (TKI) targeting EGFR, ERBB2 or ERBB4, such as AST-1306 (Cohen’s *d* = 1.71, adj. *p* = 0.14) and CI-1033 (Cohen’s *d* = 1.43, adj. *p* = 0.17) (Figure 3e). Remarkably, other screened TKIs such as pelitinib (Cohen’s *d* = 1.17, adj. *p* = 0.25) and PF-00299804 (Cohen’s *d* = 0.98, adj. *p* = 0.25) showed similar trends but did not pass our set FDR threshold of 20%. Interestingly, all CCLs with a copy number gain of EGFR showed sensitivity to TKIs independent of ancestry, but here we can reveal that other Caucasian CCLs with wild-type EGFR respond better than their Asian CCL wild-type counterparts.

Somatic driver mutations are commonly investigated as potential drug response biomarkers. It is likely that the frequency by which somatic mutations are observed in patients can be dependent on their ancestry. Consequently, we screened for enrichments of high-confidence cancer genes in CCLs in either Asian or Caucasian ancestry CCLs (Methods). We only found a handful of enriched cancer genes, namely for Asian CCLs; NF1 mutations are more abundant for GBM (adj. *p* = 0.05; Figure 3c) and mutations in MLL2 or PIK3R1 are more prevalent for COREAD (adj. *p* = 0.26 and adj. *p* = 0.21, respectively; Figure 3d). However, none of these mutations explained the ancestry-dependent variability of drug responses.

## 3. Discussion

Drug approval agencies are bound by demographics, e.g., the EMA in Europe or the FDA in the USA, and have an undeniable impact on pharmacology [17]. In order to estimate its impact on drug response, we predicted ancestry in cancer cell lines and showed a differential drug response in high-throughput drug screens. We implemented an efficient Bayesian ancestral inference which utilized ancestral genotype frequencies of SNPs and population weights in the 1000G project to successfully classify cell lines into ancestral populations, demonstrating the possibility to infer missing ancestral information in published data for both patients and cancer cell lines, even with sparse input data.

In general, there is consistency in the distributions of ancestries across cancer types, with a few exceptions. The distribution of ancestries in CCLs may reflect the demographic differences in incidence and prevalence of cancer among ethnic groups, thereby influencing the selection of CCL models. For example, a few studies have shown that Asian populations have significantly higher incidence and prevalence of ESCA and STAD as compared to Caucasian [18,19,20,21], which is consistent with our findings.

Strikingly high concordance with published data from the CCLE project supported the validity of our ancestral results and the whole analysis pipeline, building a platform for subsequent analysis and future studies. 

A univariate ANOVA analysis was conducted to assess whether ancestry can affect drug sensitivity, especially between Asian and Caucasian CCLs. The results reveal some drugs for which Asian CCLs showed higher sensitivity than Caucasian CCLs. Among the most significant associations were inhibitors targeting PI3K/mTOR signalling in GBM, especially those targeting mTOR. This was consistent with a past study involving clinical trials in solid tumours, concluding that Asian patients suffered from more severe toxicity when treated with PI3K/mTOR inhibitors than European patients who were given similar doses in solid tumours [22]. However, the direct impact of ancestry on the sensitivity of PI3K/mTOR inhibitors and the molecular mechanisms that drive the observed differential drug sensitivity have been hardly studied so far. The response of CCLs with Asian ancestry is consistent with previous studies reporting a high susceptibility of Asian patient-derived cell lines to combination treatment with nimotuzumab (EGFR inhibitor) and rapamycin (mTOR inhibitor) in GBM, which was found to be independent of their EGFR status [23].

In addition, previous studies have shown that East Asian patients are more sensitive to EGFR inhibitors, due to an observation that Asians have a higher mutational frequency of EGFR compared to Caucasians [24]. In contrast, amplifications in EGFR have been found to be more prevalent in Caucasians for cancer types such as non-small cell lung cancer [25]. Accordingly, we found that TKIs targeting EGFR, ERBB2 or ERBB4 conferred sensitivity in Caucasian CCLs in LGG. However, sensitivity was also found for Caucasian CCLs with wild-type EGFR, which is not reported thus far.

We further observed a significant number of Caucasian CCLs in the cancer types COREAD, LGG and ALL, which were more sensitive to various drugs compared to Asian CCLs, but these associations showed no enrichment of specific signalling pathways and putative drug targets. Many studies have shown the ethnic differences in the incidence and survival rates of these cancers, especially between Asian and Caucasian patients [12,13,26,27], and a few studies reported a significantly higher toxicity response to chemotherapy in COREAD Caucasian patients than Asian ones [28,29], but the cause of the differential response to targeted therapies which lies under differences in molecular profiles is still yet to be discovered by more comprehensive molecular investigations.

A limitation of this study lies in the fact that human CCLs remain simplified models which do not capture the full complexity observed in tumours, e.g., the tumour microenvironment, clonal heterogeneity or immune responses. Despite the conserved ancestral origins of CCLs, biological processes within the tumour microenvironment may differ in vivo [7], thus often hampering the generalisation of the results to patients. In addition, CCL models lack patient environments and lifestyle factors, which also can influence the sensitivity to cancer treatments [30]. It would be desirable to explore differences within subpopulations; however, we lack the statistical power due to reduced sample sizes. Thus, differential drug sensitivity analyses of subpopulations may become feasible with additional data releases in the future. Nevertheless, our findings suggest that the impact of ancestry can be partially modelled in vitro. We present a resource for ancestry and HLA subtypes of CCLs, which are shared via Cell Model Passports. This enables in vitro pharmacogenomics analyses considering demographics. In addition, we anticipate that the HLA subtype definition will become an important feature in upcoming CCLs and lymphocyte co-culturing HTS, which are currently pursued for novel immunotherapies. In summary, this study successfully elucidated the distribution of ancestries in the selection of cancer cell lines using an efficient inference pipeline and subsequent differential drug responses to PI3K/mTOR inhibitors and TKIs in GBM and LGG, respectively. We believe that this resource and subsequent findings may shape the next generation of algorithms to identify biomarkers in HTSs.

## 4. Materials and Methods

### 4.1. Data Availability

The Affymetrix SNP6.0 arrays genotyping dataset contains 1007 cancer cell lines and 884,148 SNPs ranging from chromosome 1 to chromosome 22, which are deposited in the European Genome-Phenome Archive (EGAS00001000978). For inferring ancestry, we leveraged the set of 100 ancestral SNPs from Sampson et al. [14]. Using synonyms data from Ensembl Biomart, we retrieved the genotypes of 26 ancestral SNPs, and imputed the remaining 74 SNPs.

### 4.2. Quality Control

CSV files from the Genomics of Drug Sensitivity in Cancer (GDSC) database were transformed into text (PED and MAP) and binary file sets (BED, BIM and FAM) for each chromosome using PLINK [31]. First, SNPs that had a missing rate higher than 10% and a minor allele frequency (MAF) less than 0.05 were removed. Next, positions of SNPs were compared to SNP coordinates of a legend file of the same build retrieved from the 1000 Genome Project (1000G) database (https://www.internationalgenome.org/, accessed on 20 February 2020) [15] and mismatched SNPs were removed. Finally, SNPs were checked for potential swapping of the reference strand, alternative alleles and strand flipping, and the mismatched SNPs were removed.

### 4.3. Phasing and Imputation

The quality-controlled binary file sets were converted to VCF files using PLINK. A reference genome was downloaded from the 1000G database in the form of phased VCF files and converted to BCF using BCFtools [32] and M3VCF using Minimac3 [33]. The VCF file inputs were phased using Eagle2 [34], provided the BCF reference files and genetic map from the 1000G. The phased VCF files were then imputed using Minimac4 [33] to fill the missing SNPs in each chromosome, provided the M3VCF reference genome files.

### 4.4. Inference of Ancestry

The imputed genotypes were then combined with the typed set to assemble a complete ancestral list to infer the ancestral origin of the cell lines. We calculated the probability that a cell belongs to a population given its observed ancestral genotype, the population’s genotype frequency and the population weight in the 1000G, based on Bayesian inference. The population genotype frequencies were obtained using the Ensembl API. Particularly, for a cell *Y_i_* that has the genotype *G_i_* of the ancestral SNPs independently occurring, the probability *P*(*G_i_*) that *Y_i_* belongs to a population *k* (*k* corresponds to one of 25 subpopulations in the 1000 G) is:

$$P\left({\vec{G}}_{i}\right)=\overset{100}{\underset{j=1}{\prod }}\frac{\hat{P}\left(Y_{i}=k\right)\times \left(Y_{i}=k\right)}{\hat{P}\left(G_{ij}\right)}$$

Then, the subpopulation was assigned to the cell line which had the highest corresponding probability: $arg\;max_{k}\left\{P\left({\vec{G}}_{i}\right)\right\}$. Labelling of the populations was based on the 1000G classification.

### 4.5. HLA Prediction

The imputed genotypes and the inferred ancestry of cancer cell lines (CCLs) were used to predict the genotypes of seven human leukocyte antigen (HLA) loci, including HLA-A, HLA-B, HLA-C, HLA-DPB1, HLA-DQA1, HLA-DQB1 and HLA-DRB1, at 4-digit resolution using the HIBAG algorithm [16].

### 4.6. Ancestry Biomarker Analysis

First, we combined GDSC1 and GDSC2 datasets and generated a unique drug identifier (including drug ID and dataset). Next, we removed the drugs that had extrapolated IC_50_ values (considering the maximum screening concentrations) in more than 50% of the screened CCLs. Then, we performed one-way ANOVAs across the remaining drugs and for each cancer type using ancestry as a predictor and drug response (log10(IC_50_)) as the dependent variable, adjusting for cell characteristics and growth properties as covariates. The unclassified cell lines and the cancer types that contained only one population were filtered out of the analysis. We also removed the drugs that were treated in less than 10 cell lines. To simplify the subsequent analysis and comparison to the Cancer Cell Line Encyclopedia (CCLE) dataset, we re-encoded the ancestry variable as follows: American (AMR) and European (EUR) were combined into Caucasian; East Asian (EAS) and South Asian (SAS) were combined into Asian. ANOVAs were performed in a pairwise manner between two out of the three populations. Populations that had less than three cells were not tested. The effect size was calculated as Cohen’s *d*. The *p*-values of the ANOVA tests for each cancer type were adjusted using the Benjamini–Hochberg correction.

### 4.7. Enrichment of Drug Targets

We identified enriched drug targets in drugs with differential drug response for each cancer type independently. We extracted the putative drug targets from the GDSC manifest files and for each drug target that had at least 2 significant drugs per cancer type. We subsequently tested for enrichment using a hypergeometric test for nine drug targets in total, and we adjusted the enrichment *p*-values for multiplicity using the Bonferroni adjustment method.

### 4.8. Enrichment of Somatic Driver Genes in Ancestries

We downloaded the Binary Event Matrices (BEM) from the GDSC portal (https://www.cancerrxgene.org/gdsc1000/GDSC1000_WebResources/Home.html, accessed on 13 March 2020), which contains a curated set of cancer somatic driver genes observed in both CCLs and primary tumours, from which the binary mutational status is given. We used a two-sided Fisher’s test for performing the enrichment tests. We only tested cancer types with at least one drug displaying a differential drug response, and only mutations with at least six mutated CCLs. In total, we performed 88 statistical tests, and adjusted the enrichment *p*-values for multiplicity with the Bonferroni adjustment method for each cancer type independently.

## Data Availability

The Affymetrix SNP6.0 arrays genotyping dataset contains 1007 cancer cell lines and 884,148 SNPs ranging from chromosome 1 to chromosome 22, which are deposited in the European Genome-Phenome Archive (EGAS00001000978).

## Supplementary Materials

The following are available online at https://www.mdpi.com/article/10.3390/ijms221810135/s1.

## Code Availability

All source code has been made available via https://github.com/phngbh/EIC.
